# Role of Social Support and Engagement for Cancer Survivors who Become Caregivers

**DOI:** 10.1093/geroni/igaf122.3207

**Published:** 2025-12-31

**Authors:** Natalia Babenko, William Haley, Judith Rijnhart

**Affiliations:** University of South Florida, Tampa, Florida, United States; University of South Florida, University of South Florida, Florida, United States; University of South Florida, Tampa, Florida, United States

## Abstract

Depressive symptoms significantly affect the quality of life of cancer survivors. Cancer survivors who become caregivers may experience increased caregiver burden, which may exacerbate their psychological distress. Despite these compounding challenges, the intersection of caregiving and cancer survivorship remains understudied. Social support is known to buffer against the negative psychological impact of both cancer and caregiving. This study aimed to examine the role of social engagement and social support in alleviating depressive symptoms among cancer survivors with caregiving responsibilities. A cross-sectional study was conducted with 1,388 participants from the 2021 National Study of Caregiving, focusing on caregivers aged 40 and older. The study explored the moderating and mediating roles of emotional support, service use, instrumental support, and social engagement in the relationship between cancer history and depressive symptoms. While only emotional support moderated the relationship between cancer history and depressive symptoms, the direction of effect estimates across all social support and social engagement domains consistently indicated that cancer survivors who lacked social support or engagement experienced higher depressive symptoms compared to caregivers without a history of cancer. Additionally, receiving financial help and participating in organized activities mediated the relationship between cancer history and depressive symptoms. Thus, caregivers with a history of cancer are more vulnerable to depressive symptoms in the absence of emotional support, monetary assistance, and participating in group activities. Emotional and instrumental support are critical resources for cancer survivors with caregiving responsibilities. Effective mental health strategies should maximize participation in group activities and provide emotional and financial support.

